# Anti-NMDA Receptor Encephalitis with Predominant Psychiatric Symptomatology and Diagnostic Dilemmas: A Case Report

**DOI:** 10.3390/reports9020153

**Published:** 2026-05-17

**Authors:** Djendji Siladji, Lazar Ljubotin, Jelena Amidzic, Dusan Kuljancic, Nemanja Stankovic Stevanovic

**Affiliations:** 1 Clinic of Psychiatry, University Clinical Center of Vojvodina, Hajduk Veljkova 4, 21000 Novi Sad, Serbia; 2 Faculty of Medicine, University of Novi Sad, Hajduk Vejkova 3, 21000 Novi Sad, Serbia; ljubotinl@gmail.com (L.L.); amidzicjelena0@gmail.com (J.A.)

**Keywords:** NMDAR receptor, encephalitis, immunomodulatory therapy

## Abstract

**Background and Clinical Significance**: NMDAR autoimmune encephalitis is a rare but potentially life-threatening autoimmune disorder that can be hard to recognize initially because it has nonspecific symptoms. In the early phase of the disease, clinical presentation is often dominated by psychiatric symptoms, which can be misleading. A diagnosis is established by demonstrating specific anti-NMDA receptor antibodies, with cerebrospinal fluid analysis considered the most reliable diagnostic method. Timely initiation of immunomodulatory therapy, including corticosteroids, intravenous immunoglobulins, and therapeutic plasmapheresis, significantly improves disease outcomes, while second-line therapies are used in refractory cases. **Case Presentation**: A 21-year-old female patient (M.B.) was admitted to the Psychiatry Clinic at the University Clinical Center of Vojvodina due to the sudden onset of behavioral changes, including social withdrawal, absence of verbal communication, and unusual orofacial grimacing. During hospitalization, the patient was intermittently in a state of severe psychomotor agitation and poorly communicative, with pronounced orofacial dyskinesias and involuntary tongue movements. Anti-NMDA receptor autoantibodies were detected in both serum and cerebrospinal fluid, and the patient was subsequently transferred to the Intensive Care Unit of the Neurology Clinic. Due to the lack of an adequate clinical response to pulse corticosteroid therapy, six cycles of therapeutic plasmapheresis were performed. Following this treatment, significant clinical improvement was observed. **Conclusions**: Timely recognition of this condition and a multidisciplinary approach allow for early initiation of immunomodulatory therapy and significantly improve treatment outcomes.

## 1. Introduction and Clinical Significance

Encephalitis associated with antibodies against the N-methyl-D-aspartate (NMDAR) receptor is a rare but potentially life-threatening autoimmune disorder of the central nervous system [1,2]. The pathophysiological mechanism of this autoimmune disease is the generation of autoantibodies in the brain that target the extracellular domain of the GluN1 subunit of the NMDA receptor. NMDARs are crucial for synaptic transmission, synaptic plasticity, and higher-order cognitive functions, including memory and learning. The dysfunction or internalization of these receptors due to antibody binding leads to a characteristic spectrum of neuropsychiatric manifestations [2]. The primary cause of anti-NMDAR encephalitis is NMDAR internalization without complement activation, and its malfunction leads to psychiatric and neurological symptomatology [2].

In the early phase of the disease, clinical presentation is often dominated by psychiatric symptoms, including psychomotor agitation, behavioral changes, perceptual disturbances, and delusional ideas, which frequently lead to referral for psychiatric treatment [3]. In addition, epileptic seizures, disturbances of consciousness, and autonomic nervous system dysfunction may occur [4].

Prodromal symptoms, such as elevated body temperature, headache, nausea, and vomiting, are often nonspecific and may resemble viral infections, further complicating early diagnosis [5]. Anti-NMDA receptor encephalitis most commonly occurs in young women and children; however, it can affect individuals of both sexes and all age groups [6]. Of particular importance is its association with ovarian teratomas, which are identified in a significant proportion of adult female patients, whereas the prevalence of associated neoplasms is considerably lower in children and males [7].

The diagnosis is established by demonstrating specific anti-NMDA receptor antibodies, with cerebrospinal fluid analysis considered the most reliable diagnostic method [6]. Timely initiation of immunomodulatory therapy, including corticosteroids, intravenous immunoglobulins, and therapeutic plasmapheresis, significantly improves disease outcomes, while second-line therapies are used in refractory cases [2,6].

Because psychiatric symptoms often predominate in the early phase of the disease, psychiatrists are often the first physicians to encounter patients with anti-NMDA receptor encephalitis [8]. The nonspecific nature of the initial clinical presentation, as well as the lack of response to or clinical deterioration following antipsychotic treatment, may lead to diagnostic dilemmas and delays in appropriate therapy [9]. Therefore, anti-NMDA receptor encephalitis should be considered in the differential diagnosis of acute psychotic states, particularly in young female patients with an atypical clinical course.

In this paper, we present the case of a young female patient whose illness began with predominant psychiatric symptoms and was characterized by a complex and atypical diagnostic course, highlighting the importance of early recognition of this disorder in psychiatric practice.

## 2. Case Presentation

A 21-year-old female patient (M.B.) was admitted to the Psychiatry Clinic at the University Clinical Center of Vojvodina due to the sudden onset of behavioral changes, including social withdrawal, absence of verbal communication, and unusual orofacial grimacing. According to heteroanamnestic data, she had experienced episodes of profuse vomiting on the day prior to hospitalization.

On admission, the patient was awake but markedly uncooperative. During the examination, there was a progressive increase in psychomotor tension, reaching the level of agitation. The patient continuously scanned her surroundings, giving the impression of ongoing perceptual disturbances. Verbal contact could not be established, nor was a detailed mental status examination possible. She was initially hospitalized in the Department for Schizophrenia and Other Psychotic Disorders.

Given the history of prior marijuana abuse during her high school years, as well as a confirmed positive urinary test for tetrahydrocannabinol (THC), the patient was transferred to the Department of Addictive Disorders. Due to pronounced psychomotor agitation, parenteral haloperidol therapy was initially administered, after which acute dystonic reactions rapidly developed in the form of oculogyric crises. These symptoms were resolved with the administration of biperiden, and the treatment was subsequently adjusted by replacing the typical antipsychotic with an atypical antipsychotic—olanzapine.

Over the course of her hospitalization, the patient was intermittently in a state of severe psychomotor agitation and poorly communicative, with pronounced orofacial dyskinesias and involuntary tongue movements. During physical examination, she exhibited active resistance by closing her eyes and forcefully squeezing her eyelids shut. Signs of autonomic dysfunction were recorded, including tachycardia, arterial hypertension, and a fever of up to 38 °C. Laboratory findings revealed leukocytosis (12.26 × 10^9^/L), markedly elevated creatine kinase levels (CK 6023 U/L), increased C-reactive protein (CRP 42.1 mg/L), and elevated liver transaminases (AST 183 U/L, ALT 91 U/L), Table 1.

Based on the clinical presentation and laboratory findings, malignant neuroleptic syndrome was suspected, and treatment with bromocriptine was initiated, while antipsychotic therapy was discontinued. In consultation with a neurologist, empirical antibiotic therapy with ceftriaxone was also introduced. The pupils were reactive, with mild anisocoria (left pupil smaller), while orofacial dyskinesias remained present.

A lumbar puncture was performed, and cytobiochemical analysis of the cerebrospinal fluid revealed pleocytosis (38 × 10^6^/L). Owing to the pathological cerebrospinal fluid findings, an infectious disease specialist indicated further diagnostic evaluation and treatment, and the patient was transferred to the Clinic of Infectious Diseases.

Upon admission to the Clinic of Infectious Diseases, the patient was in a severe general condition. Due to pronounced agitation, melperone was introduced into the therapeutic regimen. Brain magnetic resonance was performed, and the results were as follows: 3D T1W/3D FLAIR sagittal, T2W/FLAIR/DWI/SWI/T1W/DIR transverse, T2W coronal, 3D T1W postcontrast, T1W and FLAIR axial delayed postcontrast MR tomograms of the endocranium. The postcontrast study showed numerous artifacts that limited the evaluation to a significant extent. The study was repeated, but the delay time was extended. The extracerebral cerebrospinal fluid spaces and the ventricular system were of normal size, without pathological collections or expansions. A parietal–occipital right subcortical discretely accentuated perivascular space was observed. In the brain parenchyma supratentorially, there were no other pathological changes in morphology or signals upon examination; there were no diffusion zones, restrictions, or focal lesions of the white matter, and no manifest lesions of the brainstem or cerebellum. The foramen magnum, pontocerebellar angles and craniocervical transition were free. The sellar region and orbits were free of pathological change. Vestibulocochlear nerves, membranous cochlea and labyrinth were symmetrical, without focal pathological changes. The structures of the base of the skull included in the examination were of normal morphology, without expansive changes, and intact. Paranasal cavities and mastoid cells were free of foreign content and significant inflammatory manifestations. Normal flow at the bifurcation level of the ACC and deviated ACI was shown bilaterally, without stenotic lesions. A fetal variation in the PCA was observed on the right side. A balanced flow was observed in the VB basin, without stenotic lesions. The arteries of the Willis hexagon appeared without significant alterations in branching and signal flow. MRV findings were in order. In conclusion, brain magnetic resonance imaging revealed a discretely enlarged perivascular space in the right parieto-occipital region, without other pathological findings. Electroencephalography (EEG) in vigilance was performed, in spontaneous conduction, given that there was no cooperation during the examination and the standard activation techniques were not applied. The patient was photographed lying on a gurney. After spontaneous conduction, in legible parts of the recording, diffuse Theta/Delta activity 2–4 Hz was evident. There were no clearly formed epileptiform graph elements. In conclusion, this finding indicated a severe electrocortically manifested cerebral dysfunction throughout the recording. There were no clearly formed epileptiform graph elements. Based on the findings and the clinical presentation, the neurologist initiated high-dose parenteral pulse corticosteroid therapy with methylprednisolone (1 g/day) and anticonvulsant treatment with levetiracetam (1 g/2 times per day).

During the subsequent course of treatment, the patient developed a quantitative disturbance of consciousness and became unresponsive and soporose, with no reaction to verbal or noxious stimuli, and occasional episodes of psychomotor agitation. Anti-NMDA receptor autoantibodies were detected in both serum and cerebrospinal fluid, and the patient was subsequently transferred to the Intensive Care Unit of the Neurology Clinic. Anti-NMDA receptor antibodies were detected by identifying IgG autoantibodies against the GluN1 subunit of the NMDA receptor through cell-based assays (CBAs) using both patient cerebrospinal fluid (CSF) and serum.

On the day of transfer, the patient was awake but unable to establish verbal contact: she was afebrile, and had focal epileptic seizures manifested as oculogyric crises, without tonic–clonic convulsions. Further diagnostic evaluation for autoimmune encephalitis and paraneoplastic syndrome was continued. Computed tomography and gynecological examination did not reveal any neoplastic lesions.

Due to the lack of an adequate clinical response to pulse corticosteroid therapy, six cycles of therapeutic plasmapheresis were performed. Following this treatment, significant clinical improvement was observed: the patient became awake and communicative, with no recurrent disturbances of consciousness and no signs of pyramidal tract deficit.

The patient was discharged for home care in a stable condition, conscious, oriented, and communicative, with the following discharge therapy: levetiracetam (Lyam^®^) (UCB S.A., Brussels, Belgium) 500 mg twice daily, lorazepam 1.25 mg once daily, and palmitoylethanolamide (PEA MAX^®^) (Lepivits, Wavre, Belgium) once daily.

Two years later, pelvic magnetic resonance imaging revealed a lesion in the region of the right adnexa consistent with a mature cystic teratoma. Although this appears to be a classic, textbook example of NMDA encephalitis triggered by an ovarian teratoma at first glance, in reality, it is not that simple. The diagnosis remained unclear for a long time, especially since no textbook features of NMDA encephalitis were observed. With the initial differential diagnosis, there were many different potential treatment outcomes, which is why time was lost. The patient’s condition was first observed to be a prodrome of endogenous psychosis, then cannabis-induced psychosis, and later, as her general condition worsened and general and neurological symptoms appeared, encephalitis was suspected. However, in the initial examination of all organ systems, nothing was found that could be related to NMDA encephalitis, such as an ovarian teratoma, as described in the literature. Empirically and according to the clinical picture, a cerebrospinal fluid analysis was performed, and NMDA receptor-specific antibodies were found. Then, an appropriate diagnosis was made and treatment was initiated. This case is interesting in that the patient did not respond to the first line of treatment according to the protocol, i.e., pulse doses of corticosteroids, and plasmapheresis was started instead. When the patient had been successfully treated, recovered, and discharged from the hospital, it was concluded that this was an atypical case of anti-NMDA encephalitis because no immune-triggering entity such as an ovarian teratoma was found, and in the initial attack the patient presented a differentially diagnostically challenging case. She was regularly monitored by a neurologist, and two years after the described episode of autoimmune encephalitis, a teratoma was seen on the ovary on a routine abdominal scan. It is precisely this belated discovery of a textbook feature that we highlight in the case presented here. Even if an immunogenic focus that is classically described in textbooks is not initially found, this does not exclude the diagnosis of NMDA encephalitis, because, as seen in our case, the finding of the immunogenic focus can be delayed.

## 3. Discussion

Anti-NMDA receptor encephalitis represents a diagnostic challenge due to its heterogeneous and often nonspecific clinical presentation, particularly in the early phase of the disease, when psychiatric symptoms predominate. Under such circumstances, patients are often initially managed in psychiatric settings, which may result in delays in establishing the correct diagnosis and initiating appropriate therapy [3,8].

In the patient described, the disease began with abrupt behavioral changes, psychomotor agitation, and suspicion of a psychotic disorder, without clear neurological deficits in the initial phase. This presentation is consistent with the literature indicating that acute psychosis, agitation, and behavioral disturbances are common initial manifestations of anti-NMDA receptor encephalitis [2,6]. Additional diagnostic confusion was caused by a positive urinary test for tetrahydrocannabinol, which directed clinical reasoning toward substance-induced psychosis. The patient’s pronounced adverse reaction to the first dose of haloperidol in the form of an acute dystonic reaction, followed by a complete lack of the expected therapeutic effect after the introduction of olanzapine, prompted consideration of an organic etiology of the psychiatric disorder in the differential diagnosis.

The development of autonomic dysfunction, fever, elevated creatine kinase levels, and clinical deterioration following antipsychotic administration led to suspicion of malignant neuroleptic syndrome. The differential diagnostic dilemma between malignant neuroleptic syndrome and anti-NMDA receptor encephalitis is well-recognized and frequently described in the literature, as both conditions may involve hyperthermia, rigidity, altered consciousness, and autonomic instability [6,9]. However, the absence of the expected clinical improvement after discontinuation of antipsychotics and initiation of specific therapy for malignant neuroleptic syndrome, together with the presence of pleocytosis in the cerebrospinal fluid, indicated the need for further diagnostic evaluation for autoimmune encephalitis in this case.

Detection of anti-NMDA receptor antibodies in cerebrospinal fluid and serum represents the gold standard for establishing the diagnosis [6]. In our case, the presence of antibodies in both samples confirmed the suspicion of anti-NMDA receptor encephalitis and enabled the initiation of targeted immunomodulatory therapy. Although corticosteroids and intravenous immunoglobulins are considered first-line treatments, a proportion of patients, as in the case of our patient, do not respond adequately to this therapy [2]. Significant clinical improvement was achieved only after therapeutic plasmapheresis, which is consistent with recommendations for the management of refractory cases [6].

A particular aspect of this case report is the late detection of a mature cystic ovarian teratoma, identified two years after the initial disease episode. Although ovarian teratomas are frequently associated with anti-NMDA receptor encephalitis in young women, they may not be present or detectable at the time of diagnosis [7]. This finding highlights the importance of long-term follow-up of patients, even after clinical recovery, as well as the need for repeated diagnostic procedures to exclude a paraneoplastic etiology.

The presented case underscores the important role of psychiatrists in the early recognition of anti-NMDA receptor encephalitis. Acute psychotic symptoms, an atypical clinical course, autonomic instability, and clinical deterioration or lack of response to antipsychotic therapy should raise suspicion of an organic etiology and prompt a multidisciplinary diagnostic approach [8].

## 4. Conclusions

Anti-NMDA receptor encephalitis represents a significant diagnostic challenge, particularly in the early stages of the disease, when psychiatric symptoms often predominate in the clinical presentation. Its nonspecific presentation, as well as the lack of response or clinical deterioration following antipsychotic therapy, can lead to misdiagnosis and delays in appropriate treatment. This case highlights the importance of considering anti-NMDA receptor encephalitis in the differential diagnosis of acute psychotic episodes, especially in young patients with atypical disease courses and signs of autonomic dysfunction. Timely recognition of this condition and a multidisciplinary approach allow for early initiation of immunomodulatory therapy and significantly improve treatment outcomes.

## Figures and Tables

**Table 1:** Biochemical analysis data of the initial blood report.

Leukocytes 12.26 × 10^9^/L, Erythrocytes 3.8 × 10^12^/L, Hemoglobin 123 g/L, Hematocrit 0.348 L/L, MCV 91.3 fL, MCH 32.3 pg, MCHC 353 g/L, RDW-SD 38.9 fl, RDW-CV 0.117 R, Platelets 270 × 10^9^/L, PDW 9.4 fl, MPV 9.30 fl, P-LCR 18.8%, PCT 0.25%, Neutrophils % 74.1%, Lymphocytes % 19.2%, Monocytes % 6.0%, Eosinophils % 0.3%, Basophils % 0.4%, IG % 0.4%, Neutrophils # 9.08 × 10^9^/L, Lymphocytes # 2.36 × 10^9^/L, Monocytes # 0.73 × 10^9^/L, Eosinophils # 0.04 × 10^9^/L, Basophils # 0.05 × 10^9^/L, IG # 0.05 × 10^9^/L
Glucose 4.7 mmol/L, Urea 3.6 mmol/L, Creatinine 50 umol/L, Total bilirubin 18.5 umol/L, Direct bilirubin 4.6 umol/L, ALT 52 U/L, AST 133 U/L, GGT 20 U/L, CK 6023 U/L, CK_MB 102.0 U/L, Na 144 mmol/L, K 3.8 mmol/L, Cl 108 mmol/L, Mg 0.72 mmol/L, CRP 42.1 mg/L.

## Data Availability

The data that support the findings of this study are available from the corresponding authors (D.S. and D.K.) upon reasonable request.
